# Supplementary material for: Modeling glioblastoma heterogeneity as a dynamic network of cell states
Source: Mol Syst Biol. 2021 Sep 16;17(9):e10105. doi: 10.15252/msb.202010105 (PMC8444284; doi:10.15252/msb.202010105)
Supplement: Supplementary file 6 — Source Data for Figure 5 [file MSB-17-e10105-s004.zip › Figure5A_sourcedata/GSEA_3017/hallmarks_stateF.GseaPreranked.1621934628066/HALLMARK_EPITHELIAL_MESENCHYMAL_TRANSITION.html]

Details for gene set HALLMARK\_EPITHELIAL\_MESENCHYMAL\_TRANSITION[GSEA]

|  || Dataset | state33017 |
| Phenotype | NoPhenotypeAvailable |
| Upregulated in class | na\_pos |
| GeneSet | HALLMARK\_EPITHELIAL\_MESENCHYMAL\_TRANSITION |
| Enrichment Score (ES) | 0.2936078 |
| Normalized Enrichment Score (NES) | 1.5558313 |
| Nominal p-value | 0.03860294 |
| FDR q-value | 0.069969855 |
| FWER p-Value | 0.632 |
Table: GSEA Results Summary

  

Fig 1: Enrichment plot: HALLMARK\_EPITHELIAL\_MESENCHYMAL\_TRANSITION      
 Profile of the Running ES Score & Positions of GeneSet Members on the Rank Ordered List

  

| PROBE | GENE SYMBOL | GENE\_TITLE | RANK IN GENE LIST | RANK METRIC SCORE | RUNNING ES | CORE ENRICHMENT || 1 | PTX3 |  |  | 25 | 1.061 | 0.0274 | Yes |
| 2 | APLP1 |  |  | 28 | 1.047 | 0.0646 | Yes |
| 3 | SAT1 |  |  | 45 | 0.957 | 0.0923 | Yes |
| 4 | SPARC |  |  | 52 | 0.929 | 0.1234 | Yes |
| 5 | SPP1 |  |  | 57 | 0.898 | 0.1544 | Yes |
| 6 | ITGB5 |  |  | 80 | 0.803 | 0.1737 | Yes |
| 7 | LRP1 |  |  | 92 | 0.765 | 0.1966 | Yes |
| 8 | CDH6 |  |  | 166 | 0.635 | 0.1865 | Yes |
| 9 | PLOD2 |  |  | 183 | 0.607 | 0.2014 | Yes |
| 10 | CD44 |  |  | 191 | 0.600 | 0.2201 | Yes |
| 11 | COL5A2 |  |  | 199 | 0.589 | 0.2384 | Yes |
| 12 | HTRA1 |  |  | 229 | 0.568 | 0.2459 | Yes |
| 13 | POSTN |  |  | 296 | 0.511 | 0.2345 | Yes |
| 14 | ITGAV |  |  | 344 | 0.490 | 0.2310 | Yes |
| 15 | COL4A1 |  |  | 347 | 0.489 | 0.2479 | Yes |
| 16 | SCG2 |  |  | 354 | 0.485 | 0.2629 | Yes |
| 17 | MMP2 |  |  | 357 | 0.485 | 0.2796 | Yes |
| 18 | ID2 |  |  | 366 | 0.483 | 0.2936 | Yes |
| 19 | VCAN |  |  | 410 | 0.466 | 0.2910 | No |
| 20 | GJA1 |  |  | 495 | 0.436 | 0.2687 | No |
| 21 | TIMP3 |  |  | 516 | 0.428 | 0.2753 | No |
| 22 | MATN2 |  |  | 552 | 0.414 | 0.2744 | No |
| 23 | THY1 |  |  | 593 | 0.400 | 0.2708 | No |
| 24 | TNC |  |  | 613 | 0.393 | 0.2765 | No |
| 25 | SNTB1 |  |  | 649 | 0.386 | 0.2747 | No |
| 26 | PFN2 |  |  | 724 | 0.365 | 0.2543 | No |
| 27 | FAS |  |  | 740 | 0.361 | 0.2607 | No |
| 28 | FSTL1 |  |  | 774 | 0.352 | 0.2585 | No |
| 29 | ACTA2 |  |  | 813 | 0.344 | 0.2538 | No |
| 30 | VEGFA |  |  | 840 | 0.339 | 0.2543 | No |
| 31 | COL6A2 |  |  | 855 | 0.335 | 0.2602 | No |
| 32 | DST |  |  | 896 | 0.327 | 0.2539 | No |
| 33 | CD59 |  |  | 897 | 0.326 | 0.2658 | No |
| 34 | CADM1 |  |  | 935 | 0.320 | 0.2606 | No |
| 35 | FGF2 |  |  | 964 | 0.316 | 0.2594 | No |
| 36 | PDLIM4 |  |  | 982 | 0.313 | 0.2631 | No |
| 37 | GADD45A |  |  | 1028 | 0.305 | 0.2537 | No |
| 38 | SERPINE2 |  |  | 1030 | 0.304 | 0.2643 | No |
| 39 | LAMA2 |  |  | 1123 | 0.289 | 0.2330 | No |
| 40 | GPC1 |  |  | 1162 | 0.284 | 0.2260 | No |
| 41 | LAMA1 |  |  | 1173 | 0.283 | 0.2318 | No |
| 42 | FN1 |  |  | 1238 | 0.275 | 0.2127 | No |
| 43 | CDH11 |  |  | 1240 | 0.275 | 0.2223 | No |
| 44 | VCAM1 |  |  | 1266 | 0.272 | 0.2208 | No |
| 45 | PRRX1 |  |  | 1341 | 0.262 | 0.1967 | No |
| 46 | GEM |  |  | 1391 | 0.257 | 0.1838 | No |
| 47 | ITGA2 |  |  | 1395 | 0.257 | 0.1918 | No |
| 48 | NOTCH2 |  |  | 1420 | 0.255 | 0.1902 | No |
| 49 | EFEMP2 |  |  | 1428 | 0.255 | 0.1963 | No |
| 50 | SDC1 |  |  | 1438 | 0.254 | 0.2015 | No |
| 51 | ITGB1 |  |  | 1521 | -0.265 | 0.1738 | No |
| 52 | LGALS1 |  |  | 1799 | -0.362 | 0.0609 | No |
| 53 | TPM4 |  |  | 2039 | -0.528 | -0.0286 | No |
| 54 | TNFRSF12A |  |  | 2116 | -0.694 | -0.0378 | No |
| 55 | MCM7 |  |  | 2160 | -0.839 | -0.0268 | No |
| 56 | SERPINE1 |  |  | 2161 | -0.843 | 0.0040 | No |
| 57 | TFPI2 |  |  | 2209 | -1.026 | 0.0200 | No |
Table: GSEA details [plain text format]

  

Fig 2: HALLMARK\_EPITHELIAL\_MESENCHYMAL\_TRANSITION: Random ES distribution      
 Gene set null distribution of ES for **HALLMARK\_EPITHELIAL\_MESENCHYMAL\_TRANSITION**
